# An efficient method to enrich for knock-out and knock-in cellular clones using the CRISPR/Cas9 system

**DOI:** 10.1007/s00018-017-2524-y

**Published:** 2017-04-18

**Authors:** Francesca Niccheri, Riccardo Pecori, Silvestro G. Conticello

**Affiliations:** 1 Core Research Laboratory, Istituto Toscano Tumori, Florence, 50139 Italy; 2 Department of Oncology, Azienda Ospedaliero-Universitaria Careggi, Florence, 50139 Italy

**Keywords:** Surrogate reporter, Genome editing, Mutagenesis, Activation-induced deaminase, Class switch recombination

## Abstract

Clustered Regularly Interspaced Short Palindromic Repeats-associated protein 9 nuclease (CRISPR/Cas9) and Transcription Activator-Like Effector Nucleases (TALENs) are versatile tools for genome editing. Here we report a method to increase the frequency of Cas9-targeted cellular clones. Our method is based on a chimeric construct with a Blasticidin S Resistance gene (*bsr*) placed out-of-frame by a surrogate target sequence. End joining of the CRISPR/Cas9-induced double-strand break on the surrogate target can place the *bsr* in frame, thus providing temporary resistance to Blasticidin S: this is used to enrich for cells where Cas9 is active. By this approach, in a real experimental setting, we disrupted the *Aicda* gene in ~70% of clones from CH12F3 lymphoma cells (>40% biallelically). With the same approach we knocked in a single nucleotide to reconstruct the frame of *Aicda* in these null cells, restoring the function in ~37% of the clones (less than 10% by the standard approach). Targeting of single nucleotide changes in other genes yielded analogous results. These results support our enrichment method as an efficient tool in genome editing.

**Electronic supplementary material:**

The online version of this article (doi:10.1007/s00018-017-2524-y) contains supplementary material, which is available to authorized users.

## Introduction

The new generations of tools that have been developed in the past few years have contributed to revolutionise the landscape of genome editing: the versatility and ease of use of TALEN and CRISPR/Cas9 technologies have brought genome editing into scientific contexts where such approach was—until recently—unthinkable.

The ease of use of these genome editing tools has steadily increased since their initial application. Thus, the ability of an array of Transcription Activator-Like Effector (TALE) modules, each recognising a specific nucleotide in a double-stranded DNA context [1, 2], was harnessed to target double-strand breaks to specific DNA targets by coupling a nuclease to the TALEs [3]. Specificity and the activity of TALENs were improved, and—soon after—kits to easily produce specific TALENs were produced [4–7]. Similarly, the bacterial CRISPR immunity, in which small RNAs serve as guides and scaffolds for the targeting of the Cas9 nuclease to its DNA target [8–12], was timely exploited to generate a powerful biotechnological tool [11, 13–16]. As for TALEN technology, also CRISPR/Cas9 underwent a rapid development that greatly improved the versatility of the approach: identification of Cas9 homologues supporting different Protospacer Adjacent Motives (PAMs), optimisation for the expression/delivery of Cas9, and modifications to the nuclease activity of Cas9 [11–13, 17–23].

Despite the ease and the improved targeting offered by these genome editing tools, the targeting efficiency can still be a burdening factor, leading to laborious screenings to identify targeted cellular clones [13, 16, 24–27].

To facilitate the identification of clones in which inactivation of specific genes had occurred a number of tools and approaches have been developed. Thus, variations on the classical theme of reporter cassettes have been developed. More recently, the concept of surrogate reporters have emerged [28–38]. The function of these surrogate reporters is closely linked to the sequence-specificity of these new technologies: a surrogate target sequence, homologous to that on the gene of interest, is used to maintain out-of-frame a reporter gene, and the repair of the double-strand break induced by the nucleases leads to the activation of the reporter gene. This approach can be used to confer specific characteristics (fluorescence, cell-surface antigens, antibiotic resistance) to cells in which the nucleases—be it Zn-finger nucleases, TALENs, or Cas9—are active and the targeting of the gene of interest is more probable.

Here we report a variation of the approach in which we use a chimeric construct in which a Blasticidin S Resistance gene (*bsr*) is placed out-of-frame by a surrogate target sequence and can be used to enrich for targeted cells both for gene inactivation and—more importantly—for knock-in/mutation of mammalian cells.

## Materials and methods

### Plasmids, ssDNA donor sequences, and sequencing

Targeting of all genes was obtained using the pX330-U6-Chimeric_BB-CBh-hSpCas9, a gift from Feng Zhang (Addgene plasmid #42230 [13]). The target sequences to be used as sgRNAs were designed using the Zhang Lab’s online generator (http://crispr.mit.edu/). Oligonucleotides specific for the second exon of the *Aicda* gene, to be used either for knock-out (#1 and #2 from the list of oligonucleotides, Suppl. Table 1) or for knock-in (#3, #4), for the EGFP cds (#5, #6), and for the GTF2I gene (#7, #8) were cloned after annealing and phosphorylation into the pX330 backbone digested with BpiI (a BbsI isoschizomer). Similarly, two sgRNAs were designed to target the regions flanking the APOBEC3 *locus*. The sgRNAs (#9-#12) were cloned into the Multiplex CRISPR/Cas9 system (Kit # 1000000055) according to the protocol detailed in Sakuma et al. [39].

The plasmid to test the targeting of the sgRNA was built by inserting an adaptor (#13, #14) into the mCherry-ApoB-EGFP plasmid described in Severi and Conticello [40]. The adaptor contained BsmBI sites to allow the insertion of the surrogate targets (*Aicda* Knock-out: #15, #16; EGFP mutagenesis: #17, #18; *Aicda* Knock-in: #19, #20; A3 Knock-out: #21-#24; GTF2I mutagenesis: #25, #26). The surrogate targets are identical to the sgRNA sequences but for the addition of the PAM sequence at their 3′ end. None of the sgRNA designed to be used for mutagenesis/knock-in were able to recognise the ssDNA.

An initial construct bearing the surrogate target in between the mCherry and *bsr* coding sequences was assembled by moving the mCherry-adaptor-EGFP fragment into the pBluescript SK + backbone (digested, respectively, with NdeI/AflII and EcoRV). The EGFP coding sequence was then replaced with the *bsr* cds amplified with #27 and #28 from the pBML5 plasmid [41] (ligated into EcoRI).

Initial experiments with this construct revealed that the CMV promoter is not suitable for expression in CH12F3 cells (efficiency of the CMV promoter varies across cell lines [42]). For this reason the mCherry-adaptor-*bsr* cassette was PCR amplified (#29, #30) and transferred under the control of the β-actin promoter in the pBML5 plasmid (BglII) to create the pBSR plasmid. As a control for the enrichment experiments the empty pBML5 was used.

The ssDNA donor templates used to drive the correction of the target sequences were designed with 48nt homology arms flanking the nucleotide to be mutated/inserted, and a mutated PAM to avoid targeting by the sgRNA/Cas9 complex (EGFP mutagenesis: #31; *Aicda* Knock-in: #32; A3 Knock-out: #33; GTF2I mutagenesis: #34).

The cassette expressing the mCherry-IRES-EGFP^Y66H^ transcript was prepared by replacing the EGFP cds (BsrGI) in the mCherry-adaptor-EGFP plasmid with a mutagenised IRES-EGFP fragment obtained by PCR (#35–38) from the AID-express-puro2 plasmid [43]. A cassette for puromycin selection was inserted after the polyadenylation site (BamHI).

The genomic regions encompassing the targeted regions were amplified with the primer pairs (*Aicda*: #39, #40; EGFP mutagenesis: #41, #42; A3 Knock-out: #43–#44; GTF2I mutagenesis: #45, #46 [44]) and either sequenced directly with the primers #47–#50, or cloned into TOPO TA Cloning Kit (Invitrogen) to assess the allelic composition.

### Cell lines, transfection, and selection protocol

CH12F3-2 cells were cultured at 37 °C, 5% CO_2_, in RPMI 1640 medium (GIBCO) supplemented with 10% foetal bovine serum (FBS; Carlo Erba), 50 µM 2-mercaptoethanol, 2 mM l-glutamine (Carlo Erba), 1 mM sodium pyruvate (GE Healthcare), and penicillin/streptomycin (Carlo Erba) (RPMI complete medium). 10^7^ CH12F3 cells were transfected by electroporation using a Gene Pulser II electroporator (Biorad, Hercules, CA, USA) (Voltage = 250 V; Capacity = 500 μF; Resistance = ∞).

HEK293T cells were cultured at 37 °C, 5% CO_2_, in Dulbecco’s modified eagle medium (DMEM, EuroClone) supplemented with 10% foetal bovine serum (FBS; Carlo Erba), 2 mM l-glutamine (Carlo Erba), and 1 mM penicillin/streptomycin (Carlo Erba). Transient transfections were performed in six-well plates (5 × 10^5^ cells) using Lipofectamine LTX (Invitrogen) and GeneJuice (Novagen) according to the manufacturer’s instructions. The various plasmids (sgRNA/Cas9, pBSR, control) were transfected with a 1:1 ratio. For the knock-in 15 μg of ssDNA per well were used. Stable clones expressing the mCherry-IRES-EGFP^Y66H^ transcript were plated in 96-well plates in medium supplemented with puromycin (1.5 μg/ml).

In the targeting experiments, cells transfected with the pBSR construct or with the pBML5 control plasmid were placed under selection with Blasticidin S (BlsS, InvivoGen) (25 μg/ml for CH12F3 cells and 15 μg/ml for HEK293T cells) at 24 h of transfection. After 48 h, the antibiotic selection was removed and all cells were seeded in four 96-well plates.

Cells transfected only with sgRNA/Cas9 constructs were either left untreated or treated with puromycin (0.6 μg/ml for CH12F3-2 cells) to select transfected cells. At 48 h of puromycin treatment all cells were seeded in four 96-well plates. In the case of untreated cells, limiting dilutions in 96-well plates were performed (1 cell per well).

Colonies were picked after 10–14 days. Only wells bearing single colonies were expanded for further analysis.

Class switch recombination was induced with TGF-ß (2 ng/ml), IL4 (2 μg/ml) and anti-CD40 antibody (0.5 mg/ml) as described by Nakamura et al. [45]. After 72 h in culture CSR was assayed in stimulated cells by FACS using an anti-IgA antibody conjugated with RPE (Southern Biotech, 1:100). Flow cytometry analysis was performed on a Accuri C6 flow cytometer (BD Biosciences) in which the standard FL2 filter was replaced with a 610/20 one.

### Statistical analysis

All statistical analyses were performed using R. One-way ANOVA with Tukey’s multiple comparison test was performed to analyse the various enrichments.

## Results and discussion

To prepare the surrogate target plasmid (pBSR), we placed the coding sequences (cds) for the mCherry and for the resistance against Blasticidin S (*bsr*) under the control of the β-actin promoter, as such promoter is active in a broader range of cell lines compared to the CMV-based ones. A linker between the coding sequences places the *bsr* cds out-of-frame with the mCherry cds. The design of the linker allows the insertion of the target sequence for the sgRNA/Cas9 through BsmBI sites (in contrast to the sequence used for the sgRNA, the sequence for the surrogate target must contain the PAM sequence).

**Fig. 1:**
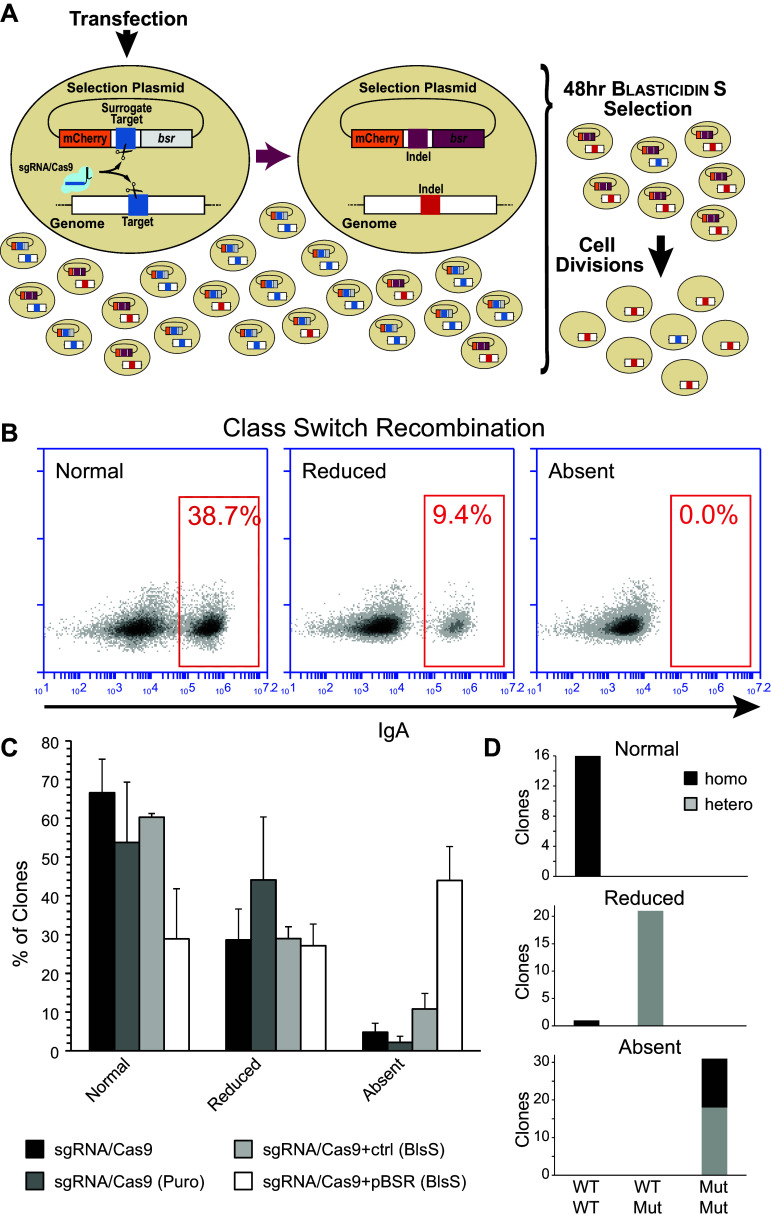
Enrichment of cells targeted by genome editing tools using the pBSR selection plasmid. **a** The selection plasmid contains a cassette in which the coding sequences for mCherry and the Blasticidin S resistance gene (*bsr*) are connected by a linker containing the target sequence (*blue*) for sgRNA/Cas9. The linker (*blue*) places the *bsr* out-of-frame (*grey*) with the mCherry. Upon cotransfection, the sgRNA/Cas9 targets the plasmid, and the frame shift resulting from the resolution of the double-strand break (*red*) allows expression of the *bsr* protein (*purple*). The acquired transient resistance to Blasticidin S will provide a proxy for the activity of the sgRNA/Cas9 in the cells, thereby allowing the selection of potentially edited cells. Removal of the antibiotic from the culture medium and limiting dilutions will enable to obtain clones targeted by the genome editing tool and devoid of the selection plasmid. **b** Representative FACS analysis of class switch recombination (CSR) in CH12F3 clones targeted for inactivation of the *Aicda* gene. The population of cells expressing IgA is gated in the* red box*. CSR levels could vary among experiments. Normal levels are indicative of those obtained from wild-type CH12F3 clones (typically an IgA(+) population of 40–60%); a CSR efficiency lower than 50% of wild-type CH12F3 was considered reduced; CSR levels lower than 0.1% were considered indicative of lack of CSR (absent). **c** Comparison of the efficiency of *Aicda* targeting by pBSR enrichment to other approaches. Samples were transfected with the sgRNA/Cas9 plasmid alone or in combination with a control plasmid or the pBSR. Cells were left untreated, or treated with puromycin (Puro) or Blasticidin S (BlsS). The bar diagram shows the percentage of clones obtained in each CSR efficiency group. The error bars indicate the SEM of at least three independent experiments. There is no statistical difference among the various groups when overall clones with impaired CSR are considered. Enrichment of CSR-absent clones using the pBSR is significantly different from all treatments (vs Cas9, *p* = 0.0006; vs Cas9(Puro), *p* = 0.001; vs Cas9 + ctrl(BlsS), *p* = 0.01). **d** The status of the *Aicda* alleles in selected clones from the pBSR sample has been assessed by sequencing of the targeted region. The plot depicts the allele composition of a number of independent clones from each CSR efficiency group. Wild-type (WT) and mutated (Mut) alleles are indicated on the *x*-axis, while the zygosity of the alleles is indicated by the colour

Cotransfection of this plasmid together with the sgRNA/Cas9 plasmids allows the selection of the cells that express a functional programmable nuclease (Fig. 1a): repair of the double-strand breaks on the surrogate target induces the formation of indels that bring the *bsr* cds in frame and provides a transient resistance to Blasticidin S.

While only a portion of indels will bring the second cds in frame, tests with a plasmid in which the *bsr* cds is replaced by an EGFP cds show that up to 70% of the transfected cells acquire the EGFP fluorescence (Suppl. Figure 1). This is likely due to the concurrent presence in the transfected cells of many targeted plasmids.

Expression of the mCherry-*bsr* chimera enables the selection of cells potentially edited at the genomic site of interest. The resistance will last as long as edited plasmids are present in the cell. In our tests we have treated cells with Blasticidin S for 48 h, but cells with a longer division time might require a longer treatment. After antibiotic treatment the surviving cells are seeded in 96-well plates in the appropriate medium. The clones obtained, having been cultured in the absence of antibiotic selection, will not contain the selection plasmid.

### Knock-out of the *Aicda* gene in CH12F3 cells

We first tested our system to obtain the inactivation of an endogenous gene in CH12F3, a murine B cell lymphoma cell line in which class switch recombination (CSR) of the immunoglobulin gene can be induced and assessed by flow cytometry [45]. The key gene that triggers CSR is Activation-Induced Deaminase, a DNA editor that targets DNA damage to the immunoglobulin gene [46]. We thus aimed to disrupt the second exon of the *Aicda* gene, which encodes for Activation-Induced Deaminase and it is present in two copies in CH12F3 cells. We selected a 20 bp sequence for use both as sgRNA and as corresponding surrogate target in the pBSR plasmid.

To test the efficiency of our system, we transfected the sgRNA/Cas9 plasmid in the presence or absence of the pBSR plasmid or of a control plasmid bearing a *bsr* cassette. After 24 h cells cotransfected with pBSR and with the control plasmid were treated with Blasticidin S to select the cells in which the CRISPR system was functional. At 72 h the antibiotic was removed and cells were either analysed for their ability to undergo CSR or plated in 96-well plates (limiting dilution was used for the samples not treated with Blasticidin S). Single colonies were picked after ~2 weeks. Since selection for cells transfected with the sgRNA/Cas9 plasmid represents a common approach to improve the targeting, we have also compared our approach to a simple selection of cells resistant to puromycin (the sgRNA/Cas9 plasmid confers puromycin resistance).

An efficient class switch recombination requires the presence of both alleles of the *Aicda* gene, as the gene is haploinsufficient and disruption of one allele substantially reduces the ability to undergo antigen-driven diversification of the antibody gene [47–49]. We thus used the ability to undergo CSR as a proxy for the efficiency of targeting of the *Aicda* gene.

We first assessed the efficiency of CSR after stimulation of the bulk population (Suppl. Figure 2). The decrease of the IgA(+) population in the cells transfected with pBSR and selected with Blasticidin S suggests that the surrogate target approach improves targeting efficiency. Nonetheless, there are two caveats that make our approach not particularly suitable for direct analysis of the whole cellular mixture. First, the short-term outcome of the combination between electroporation and antibiotic treatment—especially in cells transfected with pBSR—greatly reduces the number of viable cells. Moreover, since CSR is a stochastic event that usually involves 20–50% of the cellular population, the observed decrease in CSR could be ascribed either to successful targeting of the *Aicda* locus or to partial activation of the cellular mixture. On the other hand, selection of individual clones obtained after expression of the sgRNA/Cas9 (with or without selection) allows us to easily determine the targeting of the *Aicda* locus for each clone.

After expansion of the clones, CSR was induced and the presence of a IgA(+) population was assayed by flow cytometry. Clones were then classified according to the percentage of IgA(+) cells. Samples were binned into three CSR levels: the levels in the range of those obtained from wild-type CH12F3 cells were considered normal (typically 30–60%); a CSR efficiency lower than 50% of that from wild-type CH12F3 was considered reduced; CSR levels lower than 0.1% were considered indicative of lack of CSR (absent) (Fig. 1b).

Indeed, ~70% of the clones obtained using our approach showed an impaired CSR, compared to ~40% obtained through the other approaches (Fig. 1c). Intriguingly, the major difference between our surrogate target reporter approach and the other ones is the percentage of clones lacking IgA(+) cells altogether—indicative of biallelic targeting. The lack of difference between cells treated with antibiotics and the untreated controls is puzzling. We speculate that this might derive by the combination of high efficiency of transient expression (typically >60%) followed by death of more than >90% of electroporated cells. Under these conditions the value of antibiotic treatment for the selection of transfected cells is probably thwarted.

To confirm that the efficiency of CSR was indicative of monoallelic/biallelic targeting, we next analysed the genotype of a sample from the obtained clones by sequencing the targeted region in the second exon of the *Aicda* gene. Sequencing was performed either on the bulk of the PCR-amplified region, to obtain information on the alleles present, or on individual alleles after cloning of the PCR-amplified region in plasmids (Suppl. Figure 3).

Indeed, all clones with a normal CSR were homozygotes in the region analysed. On the other hand, except for a single clone, all clones displaying a deficit of CSR resulted in heterozygotes. In the clones with a partial reduction of CSR one of the alleles bore an inactivating deletion, while in those unable to undergo CSR both alleles bore inactivating deletions (Fig. 1d).

In a few cases, the inactivating deletions were homozygous. In one of the experiments we found a clonal 150 bp deletion in one of the alleles with three different short deletions on the other allele. While it might be interesting to assess the timing of the deletion in relation to the cell divisions, this is not possible due to the small number of clonal events identified. Intriguingly, we found the same 150 bp deletion in three independent experiments. The outcome of DNA repair—including that induced by CRISPR/Cas9—is not random, as it depends on the cellular state and on the context of the DNA lesion [e.g. 50]. Indeed—while we cannot rule out off-target effects of the sgRNA or selective advantage of the altered allele—the presence of a 10 bp homology at the boundary of the deletion suggests that repair through Microhomology-Mediated End Joining could explain the recurrence of this outcome.

It is noteworthy that none of the clones obtained after the transient treatment with the antibiotic showed mCherry fluorescence or Blasticidin S resistance. We have attempted to amplify from the selected clones a fragment of the mCherry-EGFP cassette, but we could not find evidence of its integration in the cells. We observed loss of the surrogate target reporter plasmid in all the clones obtained in the subsequent experiments. This suggests that the transient treatment is not sufficient to select for incorporation of the surrogate target plasmid.

These results suggest that the use of our surrogate target system substantially increases the chances of obtaining clones in which the target genomic sequence has been disrupted.

### Knock-in to restore an EGFP gene in HEK293T cells

**Fig. 2:**
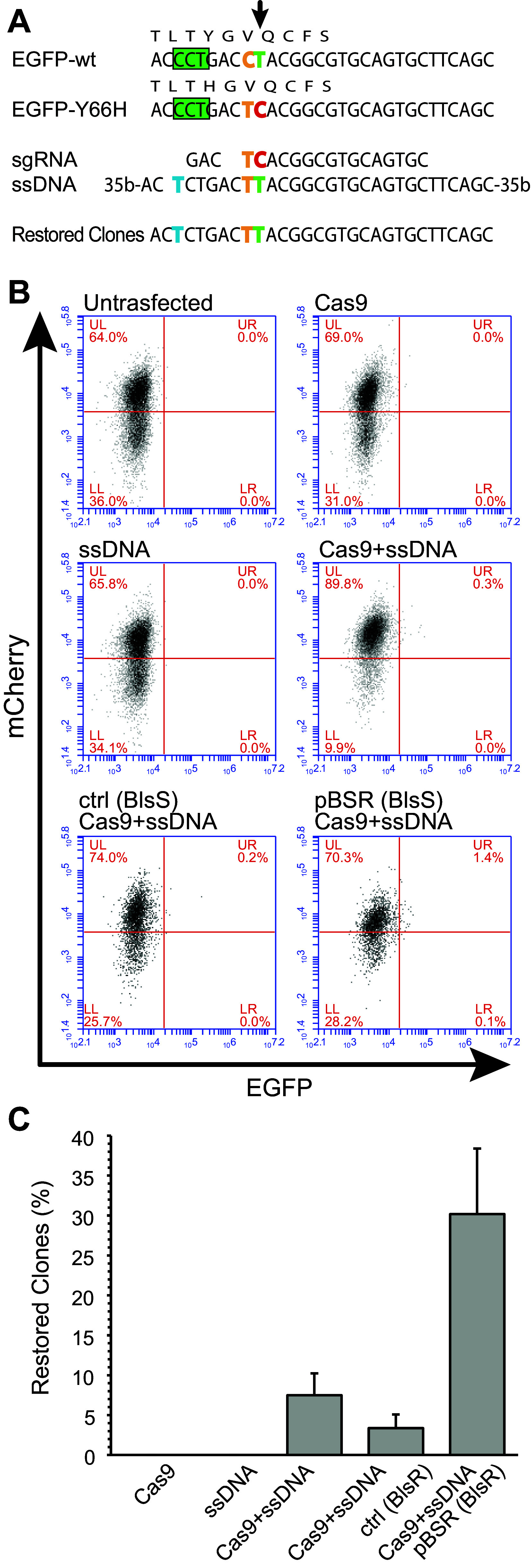
Reversion of an inactive EGFP reporter gene by knock-in. **a** A single point mutation (T>C) (*green* > *red*, *black arrow*) disables the EGFP in an mCherry-EGFP^Y66H^ reporter cassette stably transfected in a HEK293T clone. To restore the EGFP fluorescence, an sgRNA for the mutated sequence (PAM sequence indicated by the *green box*) was used in combination with a single-stranded 100nt DNA fragment (ssDNA) bearing both the restoring mutation (*green*) and a mutated PAM sequence (*blue*), to escape cleavage by Cas9. A C>T polymorphism (*yellow*) was also inserted to distinguish the sequence in mutated clones from potential contamination by wild-type EGFP. Sequencing of clones selected with our enrichment system (Restored) confirmed the knock-in of the restoring mutation. **b** Representative FACS analysis of green fluorescence restoration in mCherry-EGFP^Y66H^ expressing cells using either sgRNA/Cas9 alone (Cas9), ssDNA, sgRNA/Cas9 coupled with the ssDNA fragment (Cas9 + ssDNA), or the sgRNA/Cas9 and the ssDNA fragment together with the pBSR plasmid (pBSR(BlsR) + Cas9 + ssDNA) or with a control plasmid (ctrl(BlsR) + Cas9 + ssDNA). mCherry-EGFP^Y66H^ stable clones were transiently transfected. The cells were analysed by FACS 1 week after antibiotic selection of the pBSR-transfected samples. The percentage of cells that reacquire the green fluorescence is indicated in the upper right quadrant (UR). At least three independent experiments were performed (average population in the UR quadrant: 0% for untransfected, ssDNA, and Cas9; 0.2% for Cas9 + ssDNA; 0.1% for ctrl(BlsR) + Cas9 + ssDNA; 1.1% for pBSR(BlsR) + Cas9 + ssDNA). (c) Efficiency of knock-in in clones obtained using the different protocols. The* bar diagram* indicates the percentage of clones in which the EGFP fluorescence was restored (overall number of clones: Cas9, 45; ssDNA, 42; Cas9 + ssDNA, 131; ctrl(BlsR) + Cas9 + ssDNA, 39; pBSR(BlsR) + Cas9 + ssDNA, 70). The* error bars* indicate the SEM from at least three independent experiments. Enrichment of EGFP-restored clones using the pBSR is significantly different from all treatments (*p* < 0.05)

Having successfully used CRISPR to knock-out the *Aicda* gene in CH12F3 cells, we evaluated the utility of our system for targeted gene modification by assessing its efficiency to introduce a C>T change into an EGFP cds bearing the mutation Y66H that disrupts the fluorophore. We first obtained a stable HEK293T cell line expressing an mCherry-IRES-EGFP^Y66H^ transcript. We then selected a 20nt sequence for both the sgRNA and the pBSR surrogate plasmid. To target the mutation, we used a 100nt ssDNA donor harbouring both the desired C>T change flanked by 48nt homology regions and a mutated PAM, to avoid targeting by the sgRNA/Cas9 (Fig. 2a).

Cells were cotransfected with various combinations of the sgRNA/Cas9 and pBSR/control plasmids and the donor ssDNA. After 24 h cells transfected with pBSR and the control plasmid were treated with Blasticidin S to select those in which the sgRNA/Cas9 had been active. After 1 week of antibiotic treatment, the presence of an EGFP(+) population was assessed by flow cytometry (Fig. 2b). Overall, an increase in EGFP(+) cells was apparent in cells cotransfected with the pBSR surrogate plasmid compared to cells transfected only with sgRNA/Cas9, ssDNA, or the control plasmid.

To obtain cellular clones with the restored EGFP, cells were divided in 96-well plates (limiting dilutions were performed for cells not transfected with pBSR) after removal of the antibiotic (72 h post-transfection) and single clones were picked after 2 weeks of seeding.

As expected, sgRNA/Cas9 or ssDNA alone did not result in any EGFP(+) clone. On the other hand, association of sgRNA/Cas9 with the ssDNA donor resulted in ~7% of EGFP(+) clones and the addition of Blasticidin S selection using a control plasmid yielded ~3% of EGFP(+) clones. Use of pBR and Blasticidin S selection increased the number of EGFP(+) clones to ~30% (Fig. 2c). PCR amplification of the targeted region by Sanger sequencing confirmed the successful change of the EGFP coding sequence (Fig. 2a). Our data indicate that introduction of a targeted point mutation can be efficiently achieved using our approach.

### Knock-in to restore the *Aicda* gene in CH12F3 cells

**Fig. 3:**
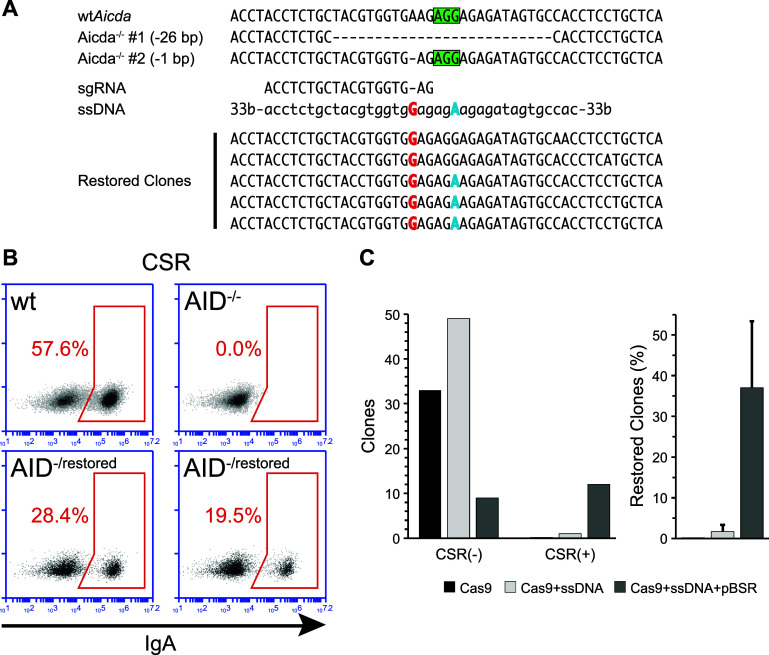
Reversion of *Aicda* ^−/−^ CH12F3 clone by knock-in. **a** Restoration of the *Aicda* gene in a clone in which both alleles of the genes were inactivated (5bis), either by a 26 bp or a 1 bp deletion. The allele with the 1 bp deletion was targeted with an sgRNA (PAM sequence indicated by the *green box*) and a single-stranded 100-mer DNA fragment (ssDNA) bearing both the base to be inserted (*red*) and a mutated PAM sequence (*blue*). The mutation on the PAM served as marker to distinguish the restored sequence from potential contamination by wild-type *Aicda*. Sequencing of clones selected with our enrichment system in which CSR was restored (restored clones) confirmed the knock-in of the restoring mutation in most clones. All clones bear the allele with the 26 bp deletion. **b** Representative FACS analysis of Class Switch Recombination (CSR) in *Aicda* ^−/−^ CH12F3 clones targeted for restoration of the *Aicda* gene. The population of cells expressing IgA is gated in the *red box*. CSR levels for wild-type (wt) and *Aicda* ^−/−^ cells are shown. Representative plots for two independent clones in which CSR was restored are shown (*Aicda* ^−/restored^). The efficiency of CSR in restored clones is diminished compared to the wild type because only one allele has been targeted. **c** Knock-in efficiency in *Aicda* ^−/−^ CH12F3 cells using either sgRNA/Cas9 alone (Cas9), sgRNA/Cas9 coupled with the ssDNA fragment (Cas9 + ssDNA), or the sgRNA/Cas9 and the ssDNA fragment together with the pBSR plasmid (Cas9 + ssDNA + pBSR). The *bar* diagram *on the left* shows the total number of clones that were either proficient or deficient for CSR (overall number of clones: Cas9, 33; Cas9 + ssDNA, 50; pBSR + Cas9 + ssDNA, 21). The *bar* diagram *on the right* shows the percentage of clones in which CSR was restored. The* error bars* indicate the SEM from three independent experiments. There is no statistical difference among the various groups

To test the feasibility of our approach also for the insertion of 1 bp on the knocked-out *Aicda* in CH12F3 cells, we targeted one of the two inactivated alleles from one of the previously obtained clones. The chosen clone presented one of the alleles inactivated with a single base deletion and the other allele with a 26 bp one (Fig. 3a).

Using the same approach used for the reactivation of the EGFP fluorophore, we used an sgRNA specific for the mutated allele, and a 100nt donor ssDNA bearing a single base insertion and a mutation on the PAM sequence. After cotransfection of combinations of the various plasmids with the ssDNA in the CH12F3 clone, we performed transient antibiotic treatment on pBSR-transfected cells before plating them. Clones were then picked after 2 weeks, expanded and CSR was induced.

Successfully targeted clones were able to perform CSR but, having only one functional allele, the efficiency of CSR was reduced compared to wild-type CH12F3 cells (Fig. 3b). As in the case of the targeted mutagenesis, the presence of the pBSR surrogate plasmid largely enhanced the targeting, with ~37% of the clones restored in three independent experiments, compared to ~2% in the absence of pBSR (Fig. 3c). The lack of statistical significance is due to one of the independent experiments in which only a single AICDA(−) clone was obtained by the pBSR approach.

Sanger sequencing of the targeted region from CSR proficient clones confirmed the insertion of the base to restore the frameshift (Fig. 3a). On the other hand, the other mutation present on the ssDNA donor—to inactivate the PAM—was present in all but two clones. This is probably due to the fact that this mutation was not under selection.

### Knock-out and knock-in of other genes

To further assess the pBSR enrichment system, we have tested its efficacy on other gene *loci*. First, we have targeted the APOBEC3 locus to obtain a 15 Kb deletion to ablate it (Suppl. Figure 4). To this aim we used two sgRNAs designed at both ends of the locus, which were cloned in tandem using the px330 plasmid [39]. A single 100-nt ssDNA was synthesised with its 5′ side homologous to the 5′ sgRNA and its 5′ flanking region, and its 3′ side homologous to the 3′ sgRNA and its 3′ flanking region. The ssDNA should facilitate the recombination between the regions flanking the two double-strand breaks (e.g. [51]). Transient transfection of the sgRNAs/Cas9 and the ssDNA coupled with either Puromycin selection or pBSR surrogate target plasmid and Blasticidin S selection was followed by seeding of all surviving cells in four 96-well plates. After growth and expansion of the cellular clones, genomic DNA was prepared and a DNA region encompassing the APOBEC3 locus was PCR amplified (black line, 280 bp) to assess the successful ablation of the entire locus. Indeed, the specific genomic fragment was amplified from many clones obtained using the pBSR enrichment, compared to none from those obtained through puromycin selection.

In another case, we have targeted the GTF2I gene to obtain a mutated gene at position L404H. This mutation is recurrent in thymic epithelial tumours [44]. To this aim, similar to what we have done to restore the EGFP and *Aicda* genes, we designed and cloned an sgRNA paired with a ssDNA specific for the desired change, and we have compared their targeting efficiency either using the pBSR enrichment approach or just selecting for transfected cells using puromycin (Suppl. Figure 4b). Even though we obtained only 7 clones after pBSR enrichment, all but one had been targeted, with most of them leading to deletions/insertions. Among these two clones was present the L404H mutation, with one being homozygous for the mutation. In comparison, none of ten clones analysed from the control experiment contained the mutated gene. It is noteworthy to note that, contrary to what was observed with CH12F3 cells, most clones appeared homozygous for the deletion/insertion. An increased representation of homozygous gene targeting has been previously observed in HEK293T cells (e.g. [52, 53]. Even though the number of analysed clones is too small to draw certainty, this could suggest that DNA repair specificities in each cell type might be the main factor leading to homozygous targeting.

## Conclusions

Whereas CRISPR/Cas9 gene targeting represents a huge step forward compared to previous tools, its efficiency is still relatively low, especially in cellular systems characterised by low transfectability. Here we describe an efficient approach to enrich for cells in which the CRISPR/Cas9 is active. Our system is suitable both for knock-out and knock-in. While the transient treatment with Blasticidin S diminishes the overall number of cells, our approach allows the selection of a substantial portion of the surviving cells targeted by the sgRNA/Cas9. The efficiency of our approach with regard to gene disruption is almost double compared to that of the standard technique, and almost five times higher when biallelic targeting is considered. Similarly, depending on the cellular system, the efficiency of knock-in is 4–20 times higher than that obtained from the standard approach. Such a high efficiency allows an easier selection of targeted clones even in the absence of an assayable phenotypic change. Compared to continuous antibiotic treatment, a transient treatment enables to select cells in which the plasmids used in the transfection are lost and—if needed—can be recycled for further targeting.

Other studies have proposed similar approaches using surrogate targets [28–38]. Yet, beside the use of Blasticidin S selection, which can be an useful addition to the available battery of surrogate target reporters, there are a few differences between these and our work, both with regard to the approach and to the conclusions.

Compared to earlier approaches, we use a simpler chimera including just mCherry and the *bsr*. This means that—if needed—other fluorescent proteins can be used in conjunction and—since antibiotic selection is gentler to cells than FACS sorting—it might be easier to obtain targeted clones.

The available approaches are either based on the resolution of the double-strand break by Non-Homologous End Joining (NHEJ) or by Homologous Recombination (HR), with the former geared more towards obtaining knock-out and the latter towards obtaining knock-in, at least theoretically. Indeed, even for obtaining knock-ins, Ren and colleagues [34] have shown a similar efficiency of the two approaches when the length of the ssDNA donor is up to 100 nt. Many factors could explain this (e.g. destruction of the cassette in the HR-based approaches during Cas9 targeting), and the relative simplicity of NHEJ-based surrogate reporter approaches could still represent a better option also for knock-in targeting. In fact, the enrichment of targeted mutagenesis by our NHEJ-based approach parallels the enrichments obtained through the published HR-based ones.

To our knowledge there is only another work, based on HR [32], in which integration of the surrogate reporter has been analysed in the obtained clones. Contrarily to what observed in that system, all our clones lost the surrogate reporter gene [mCherry(−), sensitive to BlasticidinS, and negative with regard to PCR amplification of the plasmid]. While we do not have a reason for this, we can only speculate that the homologous recombination could facilitate the integration of the plasmid, while the ends of the single double-strand break induced on the plasmid in our NHEJ-based approach tend to be repaired with each other.

Only few studies on mammalian cells have assessed the final outcome of the targeting on cellular clones [32, 33, 37, 38], but these are either HR-based or do not use an antibiotic selection. Our analysis shows that the use of surrogate reporters substantially increases the chances of biallelic targeting, even higher than what was reported in the previous ones. Nonetheless, in almost half of the targeted clones only one allele was targeted, thus suggesting that clonal selection is still a necessity.

Overall, our strategy demonstrates that surrogate reporters are a powerful option to obtain knock-out and knock-in of endogenous genes in mammalian cells, a device worth to be added to the genome editing toolbox.

### Electronic supplementary material

Below is the link to the electronic supplementary material.

Suppl. Table 1 – List of oligonucleotides used (DOCX 123 kb)

Suppl. Fig. 1 - Acquisition of EGFP fluorescence in HEK293T cells. Representative FACS analysis of HEK293T cells transiently transfected with a plasmid in which an EGFP cds was placed out-of-frame after the mCherry cds. The connecting sequence contains a sequence targeted by the sgRNA/Cas9. Cotransfection with the sgRNA/Cas9 plasmid induces the induction of indels in the linker sequence and acquisition of EGFP fluorescence due to the expression of an mCherry-EGFP chimera [31] (EPS 944 kb)

Suppl. Fig. 2 – CSR efficiency in CH12F3 population mixture. (a) Representative FACS analysis of class switch recombination (CSR) in the cellular mixture of CH12F3 cells targeted for inactivation of the *Aicda* gene. The population of cells expressing IgA is gated in the red box. CSR levels could vary among experiments. Normal levels are indicative of those obtained from wild-type CH12F3 clones. (b) Bar diagram of the CSR efficiency obtained in the cellular mixture of CH12F3 cells targeted for inactivation of the *Aicda* gene by the various approaches. The error bars indicate the SEM of three independent experiments. There is no statistical difference among the various groups when overall decrease of clones with a reduced/absent CSR is considered. The differences are not statistically significant (EPS 4021 kb)

Suppl. Fig. 3 – Sequences from CSR-deficient clones. Targeting the inactivation of the *Aicda* gene in CH12F3 cells. (a) The target for the sgRNA (red letters) and the PAM (green box) are shown on the wild-type allele. Each line represents a different allele from independent clones obtained through the selection using pBSR enrichment. Gaps indicate the presence of deletions and orange letters indicate mutations or insertions. The numbers on the side indicate how many clones with a given sequence have been identified. If identical alleles appear they originate from independent experiments. Only the mutated allele is shown for the clones with a reduced CSR (the single unmutated clone is not shown). The sequences for both alleles are shown for the clones with absent CSR. In some experiments identical sequences were obtained for more than a clone (e.g. X14, X6). While it is possible that the same outcome of the repair of the Cas9-induced DNA damage can happen (indeed, the same 150bp deletion has been observed in different experiments, on different samples, at different times), the most likely reason for this lies in the clonal expansion of a clone with a given sequence. E.g. clones marked with the black bulletpoint derive from the same experiment. (b) the DNA context flanking the recurrent 150bp deletion in the *Aicda* gene. The deleted residues are shown as dashes, and the 10bp microhomology is shown inside the box (EPS 1839 kb)

Suppl. Fig. 4 – Other gene targeting using the pBSR enrichment approach in HEK293T cells. (a) Targeting a 15Kb deletion to ablate the human APOBEC3 locus. The two sgRNAs used to target Cas9 at both ends of the locus (red) and the 100-nt ssDNA (green) to ease the recombination are shown in the schematic drawing of the APOBEC3 locus. Transient transfection of the sgRNAs/Cas9 and the ssDNA coupled with either selection or pBSR surrogate target plasmid and Blasticidin S selection was followed by seeding of the cellular mixture in four 96well-plates. After growth and expansion of the cellular clones, genomic DNA was prepared and a DNA region encompassing the APOBEC3 locus was PCR-amplified (black bracket, 280 bp) was performed to assess the successful ablation of the entire locus. The bar diagram indicates the percentage of clones in which the rearranged genomic region could be amplified (8 of the 13 clones obtained using the pBSR enrichment; none of the 10 clones obtained by puromycin selection). (b) Targeting of a single nucleotide mutation to the GTF2i locus (L404H). Wild-type and mutated GTF2I sequences are indicated, together with the sgRNA and the ssDNA used for the targeting. The arrow indicates the L440H mutation (orange). The PAM sequence (green box in the wt sequence) is disrupted in the mutated sequence (blue) to avoid retargeting by Cas9. Sequences from all pBSR-enriched clones are shown with the mutations and the deletions highlighted. The unmutated targeted base is shown in green. The bar diagram indicates the percentage of targeting outcomes obtained by pBSR enrichment [sgRNA/Cas9+pBSR(BlsR)] or by puromycin selection [sgRNA/Cas9 (Puro)] (EPS 1629 kb)
