# Supplementary material for: An efficient method to enrich for knock-out and knock-in cellular clones using the CRISPR/Cas9 system
Source: Cell Mol Life Sci. 2017 Apr 18;74(18):3413–23. doi: 10.1007/s00018-017-2524-y (PMC5544813; doi:10.1007/s00018-017-2524-y)
Supplement: Supplementary file 1 — Suppl. Table 1 – List of oligonucleotides used (DOCX 123 kb) [file 18_2017_2524_MOESM1_ESM.docx]

**Supplementary Table 1 - List of oligonucleotides**

| 1 | Aicda Knock-out sgRNA F | CACCGACCTCTGCTACGTGGTGAAG |
| --- | --- | --- |
| 2 | Aicda Knock-out sgRNA R | AAACCTTCACCACGTAGCAGAGGTC |
| 3 | Aicda Knock-in sgRNA F | CACCGTACCTCTGCTACGTGGTGAG |
| 4 | Aicda Knock-in sgRNA R | AAACCTCACCACGTAGCAGAGGTAC |
| 5 | EGFP mutagenesis sgRNA F | CACCGAGCACTGCACGCCGTGAGTC |
| 6 | EGFP mutagenesis sgRNA R | AAACGACTCACGGCGTGCAGTGCTC |
| 7 | GTF2I mutagenesis sgRNA F | CACCGGGAGAGGATATTACTTGCAA |
| 8 | GTF2I mutagenesis sgRNA R | AAACTTGCAAGTAATATCCTCTCCC |
| 9 | A3 5’ Knock-out sgRNA F | CACCGGCACACAATGCCAGACACTA |
| 10 | A3 5’ Knock-out sgRNA R | AAACTAGTGTCTGGCATTGTGTGCC |
| 11 | A3 3’ Knock-out sgRNA F | CACCGTAAGATACTGAGTTAAGGCC |
| 12 | A3 3’ Knock-out sgRNA R | AAACGGCCTTAACTCAGTATCTTAC |
| 13 | adaptor mCherry-ApoB-EGFP F | AGCTTCGTCTCTGTCGTGCCGGTACTGATGTTCTGCGAGACGG |
| 14 | adaptor mCherry-ApoB-EGFP R | AATTCCGTCTCGCAGAACATCAGTACCGGCACGACAGAGACGA |
| 15 | surrogate target for Aicda Knock-out F | GTCGACCTCTGCTACGTGGTGAAGAGG |
| 16 | surrogate target for Aicda Knock-out R | CAGACCTCTTCACCACGTAGCAGAGGT |
| 17 | surrogate target for EGFP mutagenesis F | GTCGCCTGACTCACGGCGTGCAGTGCT |
| 18 | surrogate target for EGFP mutagenesis R | CAGAAGCACTGCACGCCGTGAGTCAGG |
| 19 | surrogate target for Aicda Knock-in F | GTCGTACCTCTGCTACGTGGTGAGAGG |
| 20 | surrogate target for Aicda Knock-in R | CAGACCTCTCACCACGTAGCAGAGGTA |
| 21 | surrogate target for A3 5’ Knock-out F | GTCGGCACACAATGCCAGACACTATGG |
| 22 | surrogate target for A3 5’ Knock-out R | CAGACCATAGTGTCTGGCATTGTGTGC |
| 23 | surrogate target for A3 3’ Knock-out F | GTCGTAAGATACTGAGTTAAGGCCAGG |
| 24 | surrogate target for A3 3’ Knock-out R | CAGACCTGGCCTTAACTCAGTATCTTA |
| 25 | surrogate target for GTF2I mutagenesis F | GTCGGGAGAGGATATTACTTGCAAAGG |
| 26 | surrogate target for GTF2I mutagenesis R | cagacctTTGCAAGTAATATCCTCTCC |
| 27 | BSR cds F RP175 | AAAGAATTCTGCTGGTTATTGTGCTGTCTC |
| 28 | BSR cds R RP173 | AAAGAATTCCCACAACTAGAATGCAGTGA |
| 29 | mCherry F | AAAAAGATCTAGCGGATTCACCAT |
| 30 | bsr R | GTCGCTACTTCTACTAATTCTAGAT |
| 31 | EGFP ssDNA donor template | CACCGGCAAGCTGCCCGTGCCCTGGCCCACCCTCGTGACCACTCTGACTTACGGCGTGCAGTGCTTCAGCCGCTACCCCGACCACATGAAGCAGCACGAC |
| 32 | Aicda ssDNA donor template | ATGTCCGCTGGGCCAAGGGACGGCATGAGACCTACCTCTGCTACGTGGTGAAGAGAAGAGATAGTGCCACCTCCTGCTCACTGGACTTCGGCCACCTTCGCAA |
| 33 | A3 ssDNA donor template | CAGGAAAACACTTTTGTAATCTTGTGGTTGAGAAAGCTGGCATAAACAAGTGCGGTGGCTCATGCCTATAATCCCAGCACTTTGAGAGGCCGAGGTGGGT |
| 34 | GTF2I ssDNA donor template | CTTTTAGAAGGCCATCTACTTACGGAATTCCTCGCCTGGAGAGGATATTACATGCAAAAGAAAGGATTCGTTTTGTGATTAAGAAACATGAGCTTCTGAA |
| 35 | IRES F | AAATGTACACGAGACGCATTTCGTACTTTGGGA |
| 36 | EGFP y66h F | GTGACCACCCTGACTCACGGCGTGCAGTGC |
| 37 | EGFP y66h R | GCACTGCACGCCGTGAGTCAGGGTGGTCAC |
| 38 | EGFP R | TTTGTCGACGGCTAGTCCAGATCCAGACA |
| 39 | AICDA F | AGGGTGGGCAGGGAAGGATTTTAAAG |
| 40 | AICDA R | GAAGGTGGCCGAAGTCCAGTGA |
| 41 | EGFP F | CGTAAACGGCCACAAGTTCAG |
| 42 | EGFP R | ACTGGGTGCTCAGGTAGTGGT |
| 43 | A3 F | ACAAATGTGTGGTGGTATGTGC |
| 44 | A3 R | CTCGGCCTCTCAAAGTGCTG |
| 45 | GTF2I F | CACCGGGAGAGGATATTACTTGCAA |
| 46 | GTF2I R | AAACTTGCAAGTAATATCCTCTCCC |
| 47 | Aicda sequencing | GGCAGCCATAGCTTTAGTGTCAAC |
| 48 | EGFP sequencing | GCGATGCCACCTACGGCAA |
| 49 | A3 sequencing | ATCTTGTGGTTGAGAAAGCTGG |
| 50 | GTF2I sequencing | CTCCTGTCTGAGTCTTAGTATCCTCTT |
